# Regenerative potential of mesoporous silica nanoparticles scaffold on dental pulp and root maturation in immature dog’s teeth: a histologic and radiographic study

**DOI:** 10.1186/s12903-024-04368-6

**Published:** 2024-07-18

**Authors:** Samar Talaat, Ahmed A. Hashem, Ashraf Abu-Seida, Adel Abdel Wahed, Tarek M. Abdel Aziz

**Affiliations:** 1 Endodontic Department, Faculty of Oral and Dental Medicine, Future University in Egypt, Cairo, Egypt; 2 Department of Endodontic, Faculty of Dentistry, Ain Shams University, Cairo, Egypt; 3 Department of Surgery, Anesthesiology and Radiology, Faculty of Veterinary Medicine, Cairo University, Giza, Egypt; 4 Faculty of Dentistry, Galala University, New Galala City, Suez, Egypt

**Keywords:** Bone morphogenic protein, Immature teeth, Nanoparticles, Necotic pulps, Regenerative endodontic treatment, Revascularization

## Abstract

**Objective:**

To evaluate histologically and radiographically the potential of dog’s immature roots with apical periodontitis to regenerate after regenerative endodontic treatment using mesoporous silica nanoparticles (MSNs) with/without bone morphogenic protein (BMP-2) as scaffolds.

**Methods:**

In 4 mongrel dogs, 56 immature teeth with 96 roots were infected, resulting in necrotic pulps and periapical pathosis. According to the evaluation time (Group I = 30 days and Group II = 90 days), 90 roots were divided into two equal groups (45 roots each) and 6 roots used to replace any lost root during the procedure. The two main groups were further divided according to treatment protocol into 5 subgroups (9 roots each): blood clot (BC subgroup), mesoporous silica nanoparticles scaffold only (MSNs subgroup), mesoporous silica nanoparticles impregnated with BMP2 (MSNs + BMP2 subgroup), infected teeth without treatment (+ ve control subgroup) and normal untouched teeth (-ve control subgroup). All teeth surfaces were coated with Tincture iodine and calcium hydroxide was applied prior to treatment protocols. Then, teeth were restored with glass ionomer filling to seal the remaining part of the access cavity. Radiography evaluation of the increase in root length, root thickness and occurrence of apical closure were performed. Following the sacrifice of the two dogs at each time of evaluation, histopathological analysis was performed and included the inflammatory cells count, bone resorption, tissue ingrowth, deposition of hard tissue, and closure of the apical part. All data were statistically analyzed.

**Results:**

Compared to BC subgroup, MSNs and MSNs + BMP-2 subgroups exhibited significant higher increase in root length and thickness as well as higher vital tissue in-growth and new hard tissue formation in group II (*P* < 0.05). MSNs + BMP-2 subgroup had significant higher increase in root length and thickness as well as significant lower inflammatory cell count than MSNs subgroup in both groups (*P* < 0.05). There were no significant differences between MSNs and MSNs + BMP-2 subgroups regarding new hard tissue formation in both groups and apical closure in group I (*P* > 0.05).

**Conclusion:**

MSNs with/without BMP-2 scaffolds enabled the continuing growth of roots in immature teeth with necrotic pulps and periapical pathosis. Addition of BMP-2 to MSNs scaffold improved its outcome in regenerative endodontics.

**Clinical relevance:**

MSNs with/without BMP-2 scaffolds may alternate blood clot for regenerative endodontic treatment of immature teeth with necrotic pulps.

**Supplementary Information:**

The online version contains supplementary material available at 10.1186/s12903-024-04368-6.

## Background

Pulp necrosis slowed the growth of permanent teeth with immature roots, leaving thin teeth with weak walls that are prone to fracture [1]. Endodontic therapy for these teeth is difficult due to the number of stages involved, even with cutting-edge technologies [2–4]. Traditionally, apexification using a mineral trioxide aggregate (MTA) apical plug or long-term calcium hydroxide treatment was the preferred method. Although these treatments ease symptoms, the advantages to root development are minimal to nonexistent [5].

Regenerative endodontics is a developing science that has resulted in a “paradigm shift” in the treatment of immature teeth with ongoing root maturation and apical closure. This technique provides a distinct and novel set of physiologically based therapeutic treatments for endodontic illness [3, 6, 7].

Pulp revascularization depends upon the ability of residual pulp, apical, and periodontal stem cells to develop [3–7]. These cells are able to produce highly vascularized and conjunctive-rich live tissue. This one has the ability to colonize the accessible pulp space. These stem cells will then develop into freshly produced odontoblasts, inducing hard tissue apposition [3–7].

To refill living tissue, either the pulp chamber is filled with bioactive chemicals, or the body’s own cells are stimulated to rebuild the local tissue [8–11]. In contrast to apexification and artificial apical barrier treatments, revascularization is a biologically based therapeutic option for teeth with necrotic immature roots. Bacteria are a significant obstacle to new tissue creation, therefore effective revascularization is dependent on their absence [7, 12, 13].

Recent advances in tissue engineering have focused on three critical components of regenerative endodontic treatment: stem cells, growth factors, and scaffolds [9, 10]. Recombinant human bone morphogentic protein 2 (BMP-2) promotes dentin regeneration by increasing alkaline phosphatase activity, dentin sialo-phosphoprotein (DSPP) gene expression in vitro, and hard tissue development in vivo [14, 15].

Mesoporous silica nanoparticles (MSNs), of all known nanomaterials, are a viable drug delivery platform due to their extraordinary biocompatibility, degradability, and effective chemical and biological robustness [16]. MSNs’ unique porosity structure allows for the formation of safe environments for labile molecules and host-guest interactions, which is beneficial for medication delivery [10]. The current silica-based nanotechnology permits the synthesis of particles with various pore sizes, diameters, and structural features, allowing for fine-tuning of the final use of the Nano systems, particularly those intended to carry huge cargoes [17]. MSNs are solid materials, that contain hundreds of empty channels (mesopores) arranged in a 2D network of honeycomb-like porous structure and possess some exclusive advantages including high surface area (> 700 m2/g) and large pore volume (> 0.9 cm3/g), tunable particle size (10–1000 nm) and pore diameter (2–30 nm), tunable pore structures and physicochemical stability, uniform mesoporosity, flexible morphology, facile surface functionalisation, excellent biocompatibility and biodegradation [16, 17]. Because of their inherent tunable features, MSNs can be used as adaptable drug delivery carriers. They also provide a sturdy and rigid framework with superior chemical, thermal, and mechanical stability. MSNs and associated hybrid particles with silicon dioxide (SiO2) coatings enable straightforward modification of the resultant outer layers of Nano systems to improve biochemical stability, hence reducing side effects and potential toxicities [16, 17]. The null hypothesis states that neither the mesoporous silica nanoparticle scaffold nor its combination with BMP-2 has an impact on the histological or radiographic characteristics of tissue regeneration [18, 19].

The aim of this research was to evaluate radiographically and histologically the potential of immature dog’s teeth with apical periodontitis to regenerate after application of MSNs scaffolds with/without BMP-2.

## Materials and methods

### Sample size calculation

A previous research employed 108 dog teeth to create three equal groups (36 teeth each), each of which was further subdivided into six subgroups (6 teeth each) [6]. A total of 90 roots were sufficient as a total sample size to detect an effect size of 0.40, a power (1-β) of 80%, and a significant level of 5% (*P* < 0.05), with 45 roots representing each group. These samples represented 27 experimental roots and 18 control roots. Experimental roots were subdivided into 3 experimental subgroups which were represented by 9 roots each and the control roots were divided into positive and negative subgroups with 9 roots each. To calculate the sample size G*Power software version 3.1.9.4 was used where, 𝑓S denoted the effect size, α = 0.05, β = 0.2 and Power = 1- β = 0.8.

## Materials

### Synthesis of MSNs scaffold

In this study, we selected MSNs Mobile Composition of Matter No 41 (MCM-41, 20 g/ml). MCM-41 was synthesized using the sol-gel method with a reactant molar ratio of 1 Tetraethyl orthosilicate 98% (TEOS) (Merck KGaA, Darmstadt, Germany): 0.33 NaOH (98%) (Sigma-Aldrich, CAS: 1310-73-2, CN: S5881): 0.12 Hexa-decyltrimethyl-ammonium bromide 98% (CTAB) (Merck KGaA, Darmstadt, Germany, and CAS: 57-09-0, CN): H5882: 601.3 H_2_O [20]. Then, 0.84 g Sodium hydroxide (LOBA CHEMIE PVT. LTD., CAS: 1310-73-2ADR/PG: 8/11) was dissolved in 730 g water to make NaOH solution. After that, 2.99 g of CTAB surfactant was added and the solution was stirred until the CTAB was completely dissolved. The reaction mixture was then dropwise treated with 14.00 g of TEOS and stirred for 2 h at laboratory temperature. MCM-41 synthesized product had been washed multiple times with distilled water before being filtered and dried in an air stream. Calcination was used to eliminate surfactant molecules found in the pores of MCM-41 after drying. For 8 h, MCM-41 was calcined in an oven at a slow heating rate of 0.5 °C min^1^ from laboratory temperature to 600 °C [16]. Finally, the powder was prepared in hydrogel form by using inactive ingredient polymer gel (Nanotech Egypt for photo Electronics, Egypt) [21, 22].

### Material characterization

#### Fourier-transform infrared spectroscopy

Infrared spectra of the prepared and calcined biological materials were measured by Fourier-transform infrared (FTIR) spectroscopy on Nicolet 6700 using the attenuated total reflection (ATR) technique in the wavelength range of 4000–400 cm − 1. All spectra were recorded with a resolution of 4 cm − 1 by collecting 64 scans for a single spectrum at ambient temperature. The obtained IR data were analyzed using OMNIC, Version 8.2.0.387 software (Thermo Scientific, Thermo Fisher Scientific, Waltham, MA, USA) [22].

#### Scanning transmission electron microscopy (STEM) and energy dispersive X-ray (EDX)

Images were taken by JEOL JEM-2100 microscope operated at 200 kV. STEM-BF mode was selected for the microscope. All TEM samples were placed on a copper support grid coated with a holey carbon sheet. INCA Suite software, version 4.15 (Oxford Instruments, High Wycombe, United Kingdom), was used to process the X-ray signal generated by the samples. Sample structures were examined in STEM bright field mode using the JEOL Simple Image Viewer software, version 1.3.4. (JEOL, Tokyo, Japan) [22].

### MSNs properties

*Appearance (Color)*: White.

*Appearance (form)*: powder.

*Solubility*: soluble in toluene.

*Shape (TEM)*: rod like shape.

*Size (nm)*: length (120 ± 10) diameter (40 ± 10).

*Type*: MCM-41.

### Animal model

Four healthy mongrel dogs were purchased from AL-Fahad Trading Company for Animals (Abu Rawash, Giza, Egypt) and used in this study. The Animal Research: Reporting in Vivo Experiments Guidelines (ARRIVE) were also followed. The animals were of both sexes and their weight and age ranged between 12 and 13 kg (mean 11.5 ± 0.5) and 4–6months (mean 5.5 ± 0.5), respectively. Each dog was subjected to full physical and oral examinations by an expert veterinarian to exclude any diseased dog. The dogs were kept in the animal house at Faculty of Veterinary Medicine, Cairo University under proper conditions of nutrition, ventilation, clean environment and 12 h light/dark cycle. The animals were kept on separate kennels (1.5 m×2.5 m× 3 m) and acclimatized to housing and diet for two weeks before the experiment. They were given two meals per day (Soft food and milk) and fresh water ad libitum [12, 13].

In each dog, 14 premolars were used to sum 56 teeth constituting 96 root canals. In statistical analysis, each root was used as a unit of measurement [6]. Based on the duration of the post-treatment evaluation period, the selected teeth had been divided into two equal groups, Group I (30 days) and Group II (90 days).

### Classification of samples

This research was conducted on 90 roots with 6 extra roots to replace any lost root during the procedural steps. According to the treatment protocol, each group (*n* = 45 roots) were equally randomized and subdivided into three experimental subgroups and two control subgroups. The subgroups included blood clot only (subgroup BC), mesoporous silica nanoparticles scaffold only (subgroup MSNs), mesoporous silica scaffold impregnated with BMP-2 (subgroup MSNs + BMP2), no treatment of the infected teeth (subgroup + ve control), and normal untouched teeth (subgroup -ve control). All subgroups were represented in each dog in a randomized manner.

### Induction of periapical pathosis

After pre-medication with 0.05 mg kg-1 Atropine sulphate (ADWIA Co., 5th settlement, Cairo, Egypt) injected subcutaneously and intramuscular injection of 1 mg kg-1 Xylazine HCl (Xylaject; ADWIA Co., 5th settlement, Cairo, Egypt), general anesthesia was performed. Ketamine HCl (Keiran; EIMC Pharmaceuticals Co., Cairo, Egypt) was intravenously injected using a cannula in the cephalic vein at a dose of 5 mg kg-1 body weight to induce anesthesia. Thiopental sodium (EIPICO, Egypt) was used to maintain anesthesia at a dose of 25 mg kg-1 body weight 2.5% solution given intravenously (dose to effect) [20].

All experimental and control teeth were radiographically analyzed for incomplete root development and to form a baseline working length for future comparison. Teeth used in experimental and positive control subgroups had endodontic accesses using a high-speed hand piece (NSK hand piece, Tokyo, Japan) and size no. 2 diamond round burs (Brassler USA, Savannah, Georgia). Pulp chamber was exposed then, pulp tissues inside the canals were disrupted with a size 35 sterile H-file (Mani, Inc., Tochigi, Japan) [20]. The opening of each canal was covered with cotton and the coronal accesses were left uncovered for 3 weeks. Samples were monitored radiographically under general anesthesia after three weeks to confirm the evidence of development of periapical pathosis (radiolucent area related to the apex with interruption of the lamina dura). For pain control, Carprofen tablets (Rimadyl tab®, Zoetis, USA) were administered orally at a dose of 4.4 mg/kg once daily for 15 days [20].

Following the infection period, all the infected teeth were re-entered under general anesthesia and aseptic conditions with rubber dam isolation (Sanctuary dental dam, Sanctuary Health, SDN, BHD, Malaysia). Two roots were lost and replaced with two roots from the extra 6 roots. All teeth surfaces were coated with Tincture iodine (Biotech Pharmaceuticals PTY. LTD, Laverton North, Melbourne, Australia). File #35 was used to lightly instrument the dentinal surface and disturb the biofilm that had formed on the canal walls [20]. About 20 mL of 1.5% sodium hypochlorite were used to irrigate each canal for 5 min, followed by 20 mL of 0.9% saline solution in order to reduce cytotoxicity to apical stem cells. The irrigation needle was placed around 1 mm away from the root end [6]. The root canals were dried with sterile paper points (Meta Biomed Co. LTD, cheongwon-Gun, Chungbuk, Korea) [20]. Ultracal Ca (OH)_2_ (Ultradent Products Australia Pty Ltd, Manly, Australia) was applied as a root canal disinfectant with calcium hydroxide tip up to the previously adjusted canal length and checked radiographically [6]. Then the access cavity was sealed with a 4 mm layer of glass ionomer (Medifill^®^, Promedica, Germany) after a sterile cotton pellet was placed over the canals [6, 7].

After three weeks and under the same anesthetic and aseptic procedures the teeth were re-entered; then glass ionomer restoration was removed with a diamond stone, and the calcium hydroxide was removed using profuse saline irrigation and 20 ml of 17% EDTA (Prevest Dental Products LTD, Denpro, Digiana, Jammu, India) for 5 min per canal [6]. All experimental canals were then dried and manipulated according to the treatment modalities as follows:

### Subgroup (BC): blood clot

A hand K file size #30 was introduced at a distance of 2 mm past the apical foramen to cause bleeding to fill the canal space up to the level of the cemento-enamel junction [23]. At the cemento-enamel junction level, a resorbable matrix was used to cover the formed blood clot (CollacoteTM, Integra Life Sciences Corporation, Plainsboro, NJ, USA). White MTA (MTA Angelus, Waldir Landgraf, Londrina, PR, Brazil) was prepared according to the manufacturer’s recommendations and inserted into the canal orifice using a micro apical placement system (MAP system, Vevey, Switzerland) to make an MTA orifice plug [23]. For the MTA orifice plug inspection, teeth were radiographed. A glass ionomer filling material was used to seal the remaining section of the access cavity [6, 23].

### Subgroup (MSNs): Mesoporous silica nanoparticles scaffold

The MSNs paste was introduced inside dried canals with a sterile 20-gauge needle plastic syringe until complete filling. Then, MTA was inserted into the canal and inspected with the same way in BC subgroup where MTA orifice plug was formed. Sealing the remaining part of the access cavity was conducted with glass ionomer filling as mentioned before.

### Subgroup (MSNs + BMP2): Mesoporous silica nanoparticles scaffold impregnated with BMP-2

MSNs scaffold was drug loaded (BMP2) by the impregnation method (Nanotech Egypt for photo Electronics, Egypt) in mass ratio 1:10 of protein to nanoparticles (20ng:20 µg/ml) (MCM-41:BMP2) and prepared in hydrogel form by using inactive ingredient polymer gel. The scaffold was introduced inside the dried canals via a sterile plastic syringe with 20gauge needle until complete filling. Then, MTA orifice plug and sealing the access cavity with glass ionomer filling were performed as mentioned before.

### Subgroup (+ ve): Positive control

It included teeth with induced periapical infections that were left open and untreated [6, 23].

### Subgroup (-ve): Negative control

Teeth of this subgroup were left untouched to mature normally [6, 23].

### Radiography evaluation

Following the induction of the periapical lesion, periapical radiographs were taken and compared to follow-up radiographs taken for each subgroup at 30 and 90 days. Periapical radiographs were taken using ATECO sensor (ATECO Technology LTD, London, United Kingdom). Image-J analysis software (Image-J analysis software v1.44 National Institute of Health, USA) was used to convert digital image files to 32-bit TIFF files. To convert non-standardized pre-operative and post-operative radiographs into standardized pictures, the TurboReg plug-in (Biomedical Imaging Group, Swiss Federal Institute of Technology, Lausanne, and VD Switzerland) was utilized [6, 23].

#### Increase in root length

The length of the roots was measured in millimeters by drawing a line straight from the cemento-enamel junction to the radiographically tooth apex [6, 23].The percentage of root length increase was calculated as follows:

$$\begin{aligned} & {\text{Percentage}}\,{\text{of}}\,{\text{increase}}\,{\text{in}}\,{\text{length}} \\ & \quad =\frac{{{\text{Postoperative}}\,{\text{length}} - {\text{Preoperative}}\,{\text{length}}}}{{{\text{Preoperative}}\,{\text{length}}}} \times 100 \\ \end{aligned}$$

#### Increase in root thickness

By using the previously adjusted measurement scale, the apical third level was determined and fixed from the cemento-enamel junction. The root thickness and the pulp width were measured at this level in millimeters. Therefore, measuring the dentin thickness was by subtraction the pulp space from the whole root thickness [6, 23]. Dentin thickness = root thickness – pulp width.

$$\begin{aligned}& {\text{Percentage}}\,{\text{of}}\,{\text{increase}}\,{\text{in}}\,{\text{thickness}} \\ & \quad = {\kern 1pt} \frac{{{\text{Postoperative}}\,{\text{thickness}} - {\text{Preoperative}}\,{\text{thickness}}}}{{{\text{Preoperative}}\,{\text{thickness}}}} \times 100 \\ \end{aligned}$$

#### Decrease in apical diameter

The apical foramen’s diameter was measured in millimeters before and after using the preset measurement scale [6, 23]. Calculating the apical closure percent change was as follows.

$$\begin{aligned}& {\text{Percentage}}\,{\text{of}}\,{\text{apical}}\,{\text{closure}} \\ & \quad = \frac{{{\text{Preoperative}}\,{\text{apical}}\,{\text{diameter}} - {\text{Postoperative}}\,{\text{apical}}\,{\text{diameter}}}}{{{\text{Preoperative}}\,{\text{apical}}\,{\text{diameter}}}} \times 100 \\ \end{aligned}$$

### Histopathology evaluation

The experimental dogs were sacrificed via an anesthetic overdose (Thiopental sodium) based on the post-treatment evaluation period (2 dogs after each evaluation period). The teeth with the surrounding bone block were sawed and inserted in 10% formalin buffered solution for fixation. Decalcification was performed by immersion in 17% EDTA solution for 120 days. Sectioning of the decalcified blocks was performed at bucco-lingual direction into 6 m thickness. Hematoxylin and eosin stain was used to stain these sections. The stained sections were examined histopathologically.

#### Inflammatory cell count in the periapical tissues

It was conducted according to Tawfik et al. [6]. Briefly, three representative fields were examined at X200 magnification for each slide. Prior to calculation, binary thresholds of the specified color-coded inflammatory cells were completed. The total number of cells was then counted as a factor of 103.

#### Bone/Root resorption

It was conducted according to Tawfik et al. [6]. Score 0: There was no sign of resorption. Score 1: osteoclasts, Howship’s lacunae, and resorption regions were evident.

#### Presence of vital tissue within the pulp space

It was conducted according to Tawfik et al. [6]. Score 0: No evident of tissue ingrowth was evident inside the canal space. Score 1: Tissue in-growth was evident into the canal’s apical third. Score 2: Tissue in-growth was evident extending to the canal’s middle third. Score 3: Tissue in-growth was evident extending to the canal’s cervical third.

### New hard tissue presence or absence

#### Qualitative analysis

It was conducted according to Tawfik et al. [6]. Briefly, hard structure histological identification criteria included cementum, Haversian canals with the osteocyte-like cells, Sharpey’s fiber and presence of oedema as well as inflammatory cells; lymphocytes.

#### Quantitative analysis

It was conducted according to Tawfik et al. [6]. Briefly, Score 0: Absence of new hard tissue formation, Score 1: Partial formation of new hard tissues and Score 2: Complete formation of new hard tissues.

#### Apical closure

It was conducted according to Tawfik et al. [6]. Briefly, Score 0: Apical closure was not evident and Score 1: Apical closure was evident.

### Statistical analysis

The normality of numerical data was investigated by checking the distribution of data and applying normality tests (Kolmogorov-Smirnov and Shapiro-Wilk tests). Except for the number of inflammatory cells, which had a non-parametric distribution, all data proved a parametric distribution. For parametric data, two-way ANOVA and for non- parametric data, Kruskal-Wallis Dunn’s test were used for pairwise comparisons. Chi-square test or Fisher’s Exact test when applicable were used for comparisons related to qualitative data. The significance level was set at *P* ≤ 0.05. Statistical analysis was performed with IBM SPSS (SPSS: Statistical Packages for the Social Sciences 19.0, IBM, USA) Statistics for Windows (Version 23.0. Armonk, NY: IBM Corp).

## Results

### Radiography findings

#### Increase in root length

**Fig. 1:**
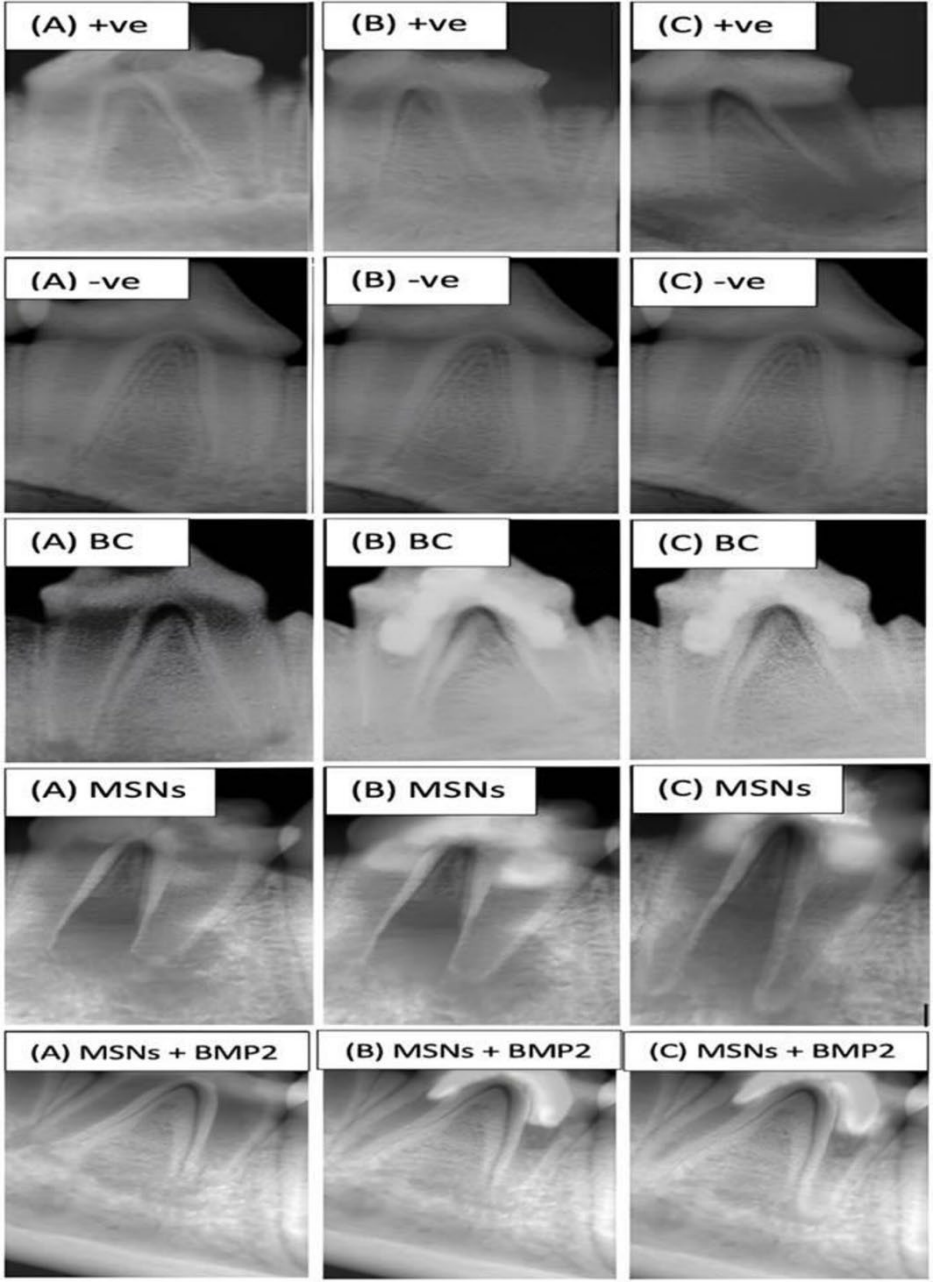
Representative radiographs of all subgroups showing changes in root length, root thickness and apical diameter at pre-operative (**A**), one month (**B**) and three months (**C**)

After one-month, MSNs + BMP-2 and negative control subgroups showed the highest statistically significant values (*P* < 0.001). with no difference between each other (*P* > 0.05). After three months, negative control subgroup showed the highest significant mean percentage increase in root length followed by MSNs + BMP-2 subgroup (*P* < 0.0001). There were statistically significant differences between all subgroups (*P* < 0.001) as shown in (Fig. 1; Table 1).

**Table 1:** The mean, standard deviation (SD) values and results of two-way ANOVA test for comparison between percentage increase in root lengths (%) in all groups and subgroups

Subgroups	Groups			
	1 month		3 months	
	Mean	SD	Mean	SD
Blood clot	7.8^C^	1.3	14^D^	1.3
Mesoporous silica nanoparticles	8.6^B^	1	16.5^C^	1.1
Mesoporous silica nanoparticles + BMP-2	11.9^A^	1.4	20.4^B^	1.2
Positive control	0^D^	0	0^E^	0
Negative control	12.3^A^	0.3	23.8^A^	0.3
P-value	< 0.001*		< 0.001*	
Effect size (Partial eta squared)	0.92		0.975	

* Significant at *P* ≤ 0.05, Different superscript letters in the same column indicate statistically significant difference between subgroups

#### Increase in root thickness

After one and three months, a statistically significant difference existed between subgroups (P-value < 0.001). The negative control group showed the highest significant mean percentage followed by MSNs + BMP-2 subgroup as shown in (Table 2; Fig. 1).

**Table 2:** The mean, standard deviation (SD) values and results of two-way ANOVA test for comparison between percentage increase in root thickness (%) in all groups and subgroups

Subgroups	Groups			
	1 month		3 months	
	Mean	SD	Mean	SD
Blood clot	7.3^D^	1.2	8.8^D^	1.2
Mesoporous silica nanoparticles jjjjjnanoparticlesnanoparticles nanoparticles	8.3^C^	1.2	10^C^	1.2
Mesoporous silica nanoparticles + BMP-2	9.2^B^	1	12.5^B^	0.5
Positive control	0^E^	0	0^E^	0
Negative control	11.9^A^	1.6	15^A^	1.4
P-value	< 0.001*		< 0.001*	
Effect size *(Partial eta squared)*	0.887		0.928	

* Significant at *P* ≤ 0.05, Different superscript letters in the same column indicate statistically significant difference between subgroups

#### Increase in apical closure

After one-month, negative control and MSNs + BMP-2 subgroups exhibited the highest significant apical closure with no difference between both of them. After three months, negative control subgroup showed the highest significant mean percentage increase in apical closure followed by MSNs + BMP-2 (*P* < 0.001). There were statistically significant differences between all subgroups as shown in (Table 3; Fig. 1).

**Table 3:** The mean, standard deviation (SD) values and results of two-way ANOVA test for comparison of percentage increase in apical closures (%) in all groups and subgroups

Subgroups	Groups			
	1 month		3 months	
	Mean	SD	Mean	SD
Blood clot	18^B^	1.5	29.1^D^	2.2
Mesoporous silica nanoparticles	19.2^B^	2	31.7^C^	2.6
Mesoporous silica nanoparticles + BMP-2	20.5^AB^	2.5	34.9^B^	1.8
Positive control	0^C^	0	0^E^	0
Negative control	22^A^	1.6	37.1^A^	4.3
P -value	< 0.001*		< 0.001*	
Effect size *(Partial eta squared)*	0.88		0.954	

* Significant at *P* ≤ 0.05, Different superscript letters in the same column indicate statistically significant difference between subgroups

### Histopathology findings

#### Inflammatory cell count

**Fig. 2:**
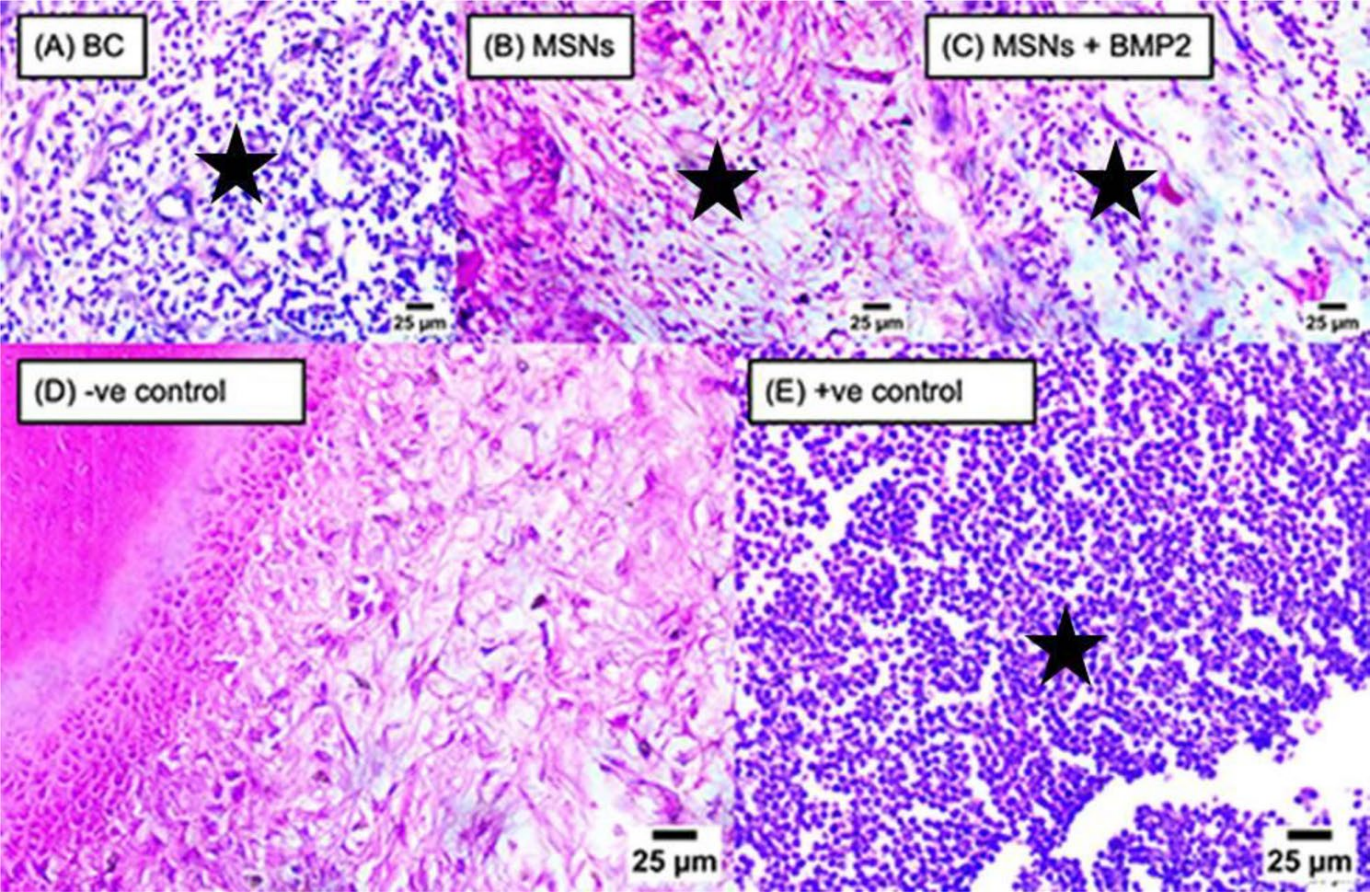
Representative photomicrographs of all subgroups showing various numbers of mononuclear inflammatory cells infiltration (black stars) after 3-months (400X, H&E)

After one month and three months, positive control subgroup showed the highest median inflammatory cell count and the negative control subgroup showed the lowest median inflammatory cell counts followed by MSNs + BMP-2 subgroup (*P* < 0.001) as shown in (Table 4; Fig. 2).

**Table 4:** Descriptive statistics and results of Kruskal-Wallis test for comparison between inflammatory cell counts in all groups and subgroups

Subgroups	Groups			
	1 month		3 months	
	Median (Range)	Mean (SD)	Median (Range)	Mean (SD)
Blood clot	483.3 (400-534.3)^B^	480.9 (50.7)	34 (12–314)^B^	136.7 (155)
Mesoporous silica nanoparticles	448.7 (368.7-510.3)^B^	448.7 (58.3)	32.3 (18–183)^B^	61.1 (63.5)
Mesoporous silica nanoparticles + BMP-2 BMP-2	343.3 (286.7–380)^C^	343.7 (35.7)	18.8 (12.7–22.7)^C^	18.4 (4.1)
Positive control	532 (518.7-602.7)^A^	544.7 (34.1)	670 (633.3-706.7)^A^	683.3 (21.5)
Negative control	14.3 (13.7–18.7)^D^	15.1 (2)	11 (9.3–11.7)^C^	10.6 (1)
*P* -value	< 0.001*		< 0.001*	
Effect size *(Eta squared)*	0.854		0.879	

* Significant at *P* ≤ 0.05, Different superscript letters in the same column indicate statistically significant difference between subgroups

#### Bone resorption

**Fig. 3:**
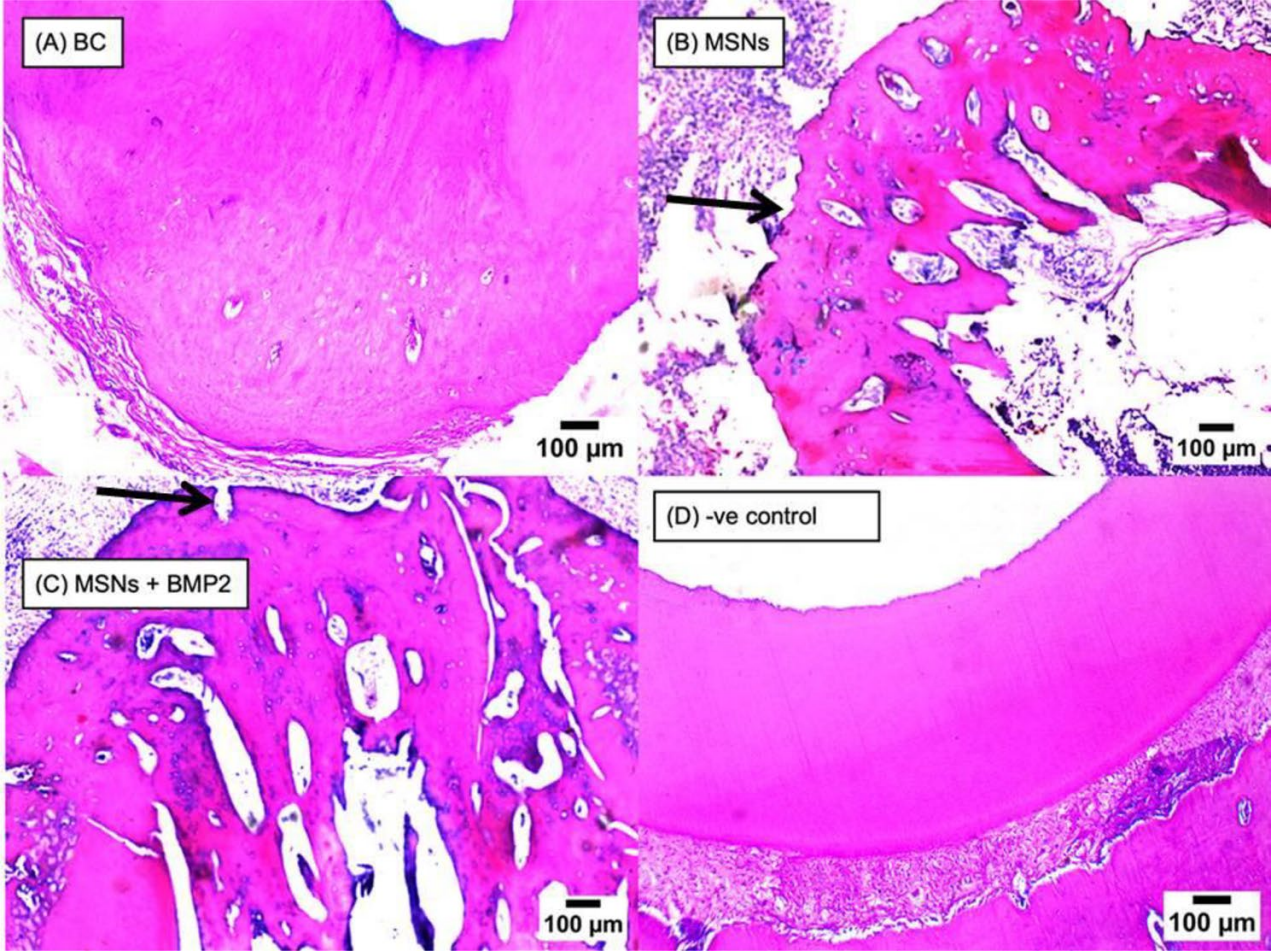
Representative photomicrographs of all subgroups showing apical root resorption (black arrows) after 3-months (H&E)

After one month and three months, the positive control subgroup had the highest significant prevalence of resorption (*P* < 0.001). MSNs and MSNs + BMP-2 subgroups showed significant lower prevalence of resorption (*P* < 0.001). While the negative control subgroup revealed no resorption. The difference across subgroups was statistically significant as shown in (Table 5; Fig. 3).

**Table 5:** The frequencies (N), percentages (%) and results of Chi-square as well as Fisher’s Exact test for comparison between prevalence of bone resorption at different times within each subgroup

Time	Blood clot		Mesoporous silica		Mesoporous silica + BMP-2		Positive control		Negative control	
	*N*	%	*N*	%	*N*	%	*N*	%	*N*	%
1 month	7	77.8	5	55.6	2	22.2	8	88.9	0	0
3 months	5	55.6	2	22.2	1	11.1	9	100	0	0
P-value	0.371		0.219		0.030		1		Not computed	
Effect size *(v)*	0.275		0.251		0.338		0.209			

* Significant at *P* ≤ 0.05

#### The nature and extent of tissue in-growth

**Fig. 4:**
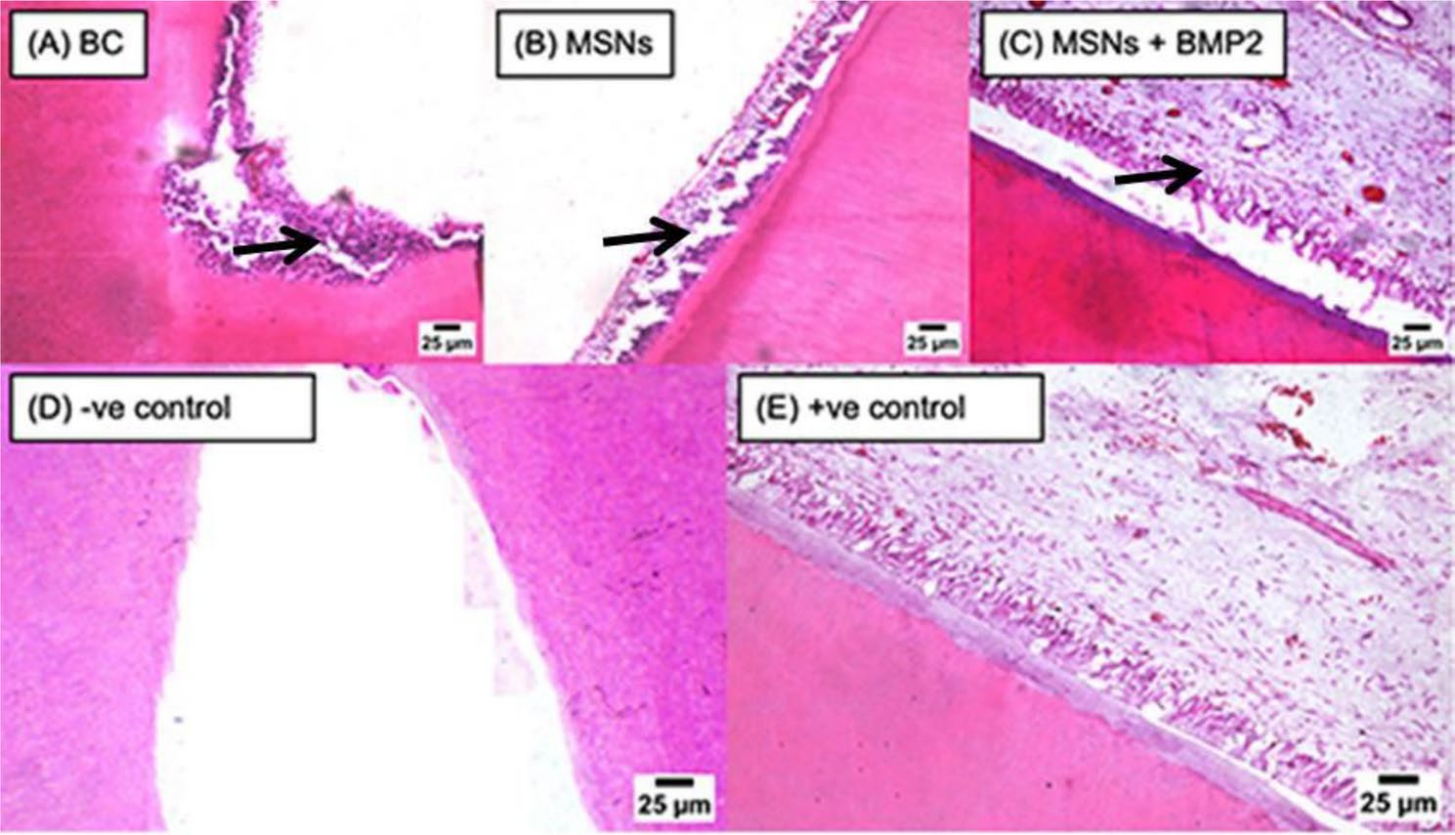
Representative photomicrographs of all subgroups showing connective tissue in growth inside the pulp cavity reaching the apical third (black arrow) in BC subgroup (**A**), reaching middle third (black arrow) of the root canal in MSNs subgroup (**B**), and reaching coronal third (black arrow) of the root canal in MSNs + BMP2 subgroup (**C**)

In some samples, histopathology examination for subgroup (BC) revealed connective tissue in-growth. In nature, this tissue appeared like periodontal connective tissue, with varied degrees of inflammatory cells infiltration and evident angiogenic activity. MSNs subgroup revealed connective tissue in-growth within the pulp space. This tissue was similar to pulp tissue in nature, with varied degrees of inflammatory cells infiltration and notable angiogenic activity. A layer of odontoblast-like cells undergoing differentiation could also be seen opposite to a predentin layer as shown in Fig. 4.

After one-month, the negative control subgroup revealed the statistically significant highest median score of tissue in-growth followed by MSNs and MSNs + BMP-2 subgroups (*P* < 0.001); both subgroups exhibited statistically significant lower median scores (*P* < 0.001). After three months, there was no statistically significant difference between MSNs + BMP-2 and negative control subgroups (*P* > 0.05); both subgroups showed the highest median scores as shown in Table 6.

**Table 6:** Descriptive statistics and results of Kruskal-Wallis test for comparison between vital tissue scores in all groups and subgroups

Subgroups	Groups			
	1 month		3 months	
	Median	Range	Median	Range
Blood clot	1^C^	0–1	1^C^	0–2
Mesoporous silica nanoparticles	2^B^	1–2	2^B^	1–3
Mesoporous silica particles + BMP-2	2^B^	1–3	3^A^	2–3
Positive control	0^D^	0–0	0^D^	0–1
Negative control	3^A^	3–3	3^A^	3–3
P-value	< 0.001*		< 0.001*	
Effect size *(Eta squared)*	0.865		0.875	

* Significant at *P* ≤ 0.05, Different superscript letters in the same column indicate statistically significant difference between subgroups

#### Formation of mineralized hard tissue

Subgroup (BC) showed apparently layer of apical hard tissue formation at the inner side of the dentin. The tissue had regular outline and variable thickness as well as a thin layer of cementoid tissue was evident covering it that gave the appearance of cementum-like tissue. There were cementoblast-like cells and cementocyte-like cells. However, empty lacunae with degenerated cementocyte-like cells were observed.

Regarding subgroups (MSNs) and (MSNs + BMP-2), apical hard tissue formation was observed on the internal radicular dentin. Apparently large areas of mineralized tissue that resembled osteodentin covered with a layer of predentin were detected. Inside the mineralized tissue, odontoblast-like cells were entrapped. Furthermore, tubular dentin was also found. In addition, odontoblast-like cells were seen opposing the predentin layer.

After one-month, negative control subgroup showed the highest median hard tissue formation. Other subgroups had no significant difference between them (*P* > 0.05). After three months, there were no statistically significant differences between the MSNs, MSNs + BMP-2, and negative control subgroups (*P* > 0.05). The positive control subgroup had the statistically lowest median score (*P* < 0.001) as shown in Table 7.

**Table 7:** Descriptive statistics and results of Kruskal-Wallis test for comparison between new hard tissue scores in all groups and subgroups

Subgroups	Groups			
	1 month		3 months	
	Median	Range	Median	Range
Blood clot	1^B^	0–2	1^B^	0–2
Mesoporous silica nanoparticles	1^B^	0–2	2^A^	1–2
Mesoporous silica nanoparticles + BMP-2	1^B^	1–2	2^A^	1–2
Positive control	0^C^	0–0	0^C^	0–0
Negative control	2^A^	2–2	2^A^	2–2
P-value	< 0.001*		< 0.001*	
Effect size *(Eta squared)*	0.615		0.652	

* Significant at *P* ≤ 0.05, Different superscript letters in the same column indicate statistically significant difference between subgroups

#### Apical closure

**Fig. 5:**
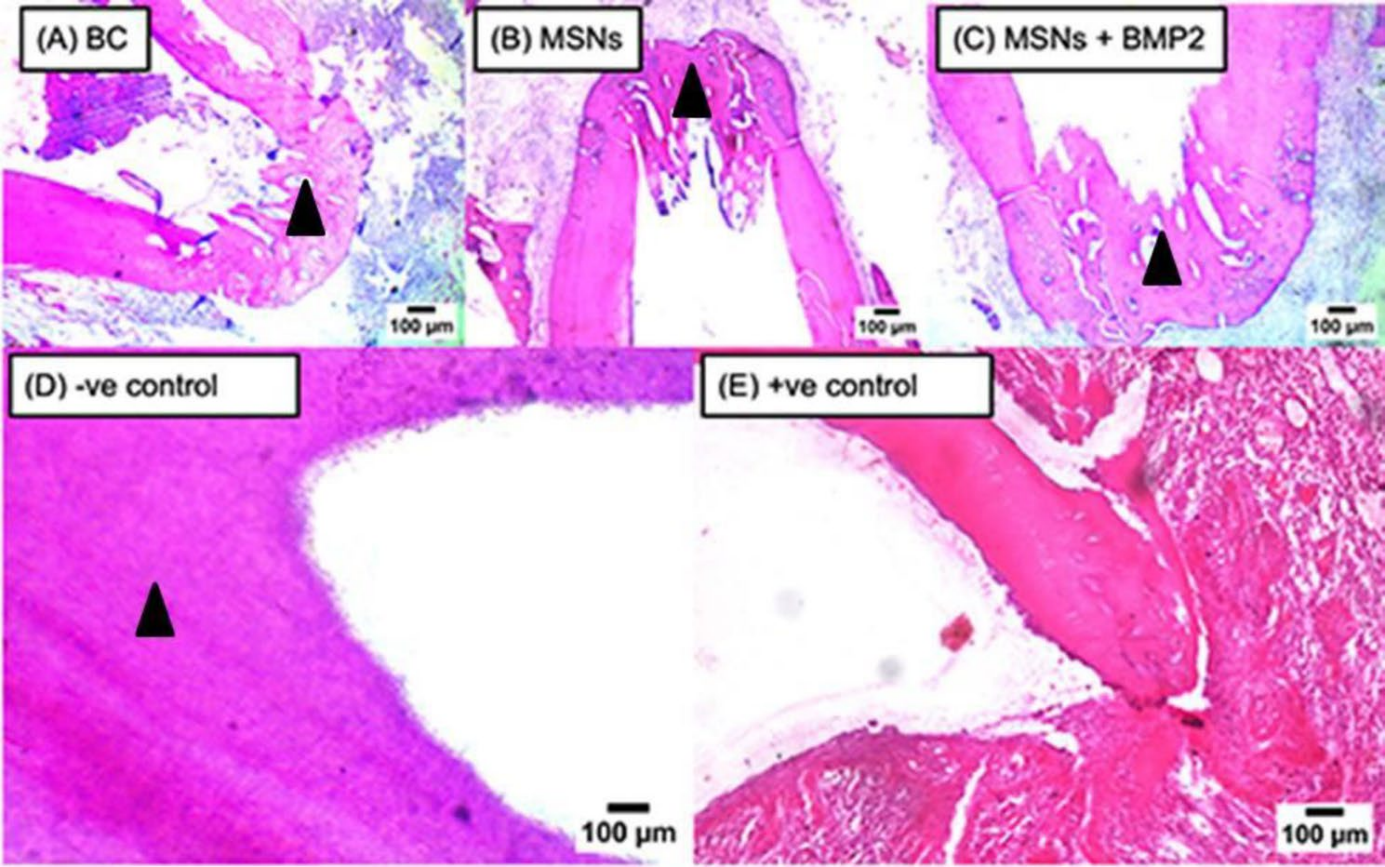
Representative photomicrographs of all subgroups showing apical closure (arrow heads) in BC subgroup (**A**), MSNs subgroup (**B**), MSNs + BMP2 subgroup (**C**) and negative control subgroup (**D**). Notice absence of any sign of apical closure in positive control subgroup (**E**)

After one and three months, negative control subgroup had the highest apical closure then MSNs + BMP-2 subgroup followed by MSNs (*P* < 0.001) as shown in (Table 8; Fig. 5).

**Table 8:** The frequencies (N), percentages (%) and results of Chi-square as well as Fisher’s Exact test for comparison between prevalence of apical closure at different times within each subgroup

Time	Blood clot		Mesoporous silica		Mesoporous silica + BMP-2		Positive control		Negative control	
	*N*	%	*N*	%	*N*	%	*n*	%	*N*	%
1 month	2	22.2	3	33.3	5	55.6	0	0	8	88.9
3 months	4	44.4	7	77.8	8	88.9	0	0	9	100
P-value	0.667		0.099		0.036		Not computed		0.478	
Effect size *(v)*	0.177		0.251		0.103				0.302	

* Significant at *P* < 0.05

## Discussion

Regenerative endodontic treatment is a biologically based technique that has recently gained popularity for treatment of immature and mature teeth with necrotic pulps [12, 24, 25]. Injectable scaffold is one of the treatment options for the regenerative endodontic triad. The aim behind integrating an injectable scaffold, hydrogel containing growth factors such as BMP, and a medication delivery method was to accelerate the regeneration process. Because of its biosafety and good protein drug inertness, the hydrogel acts as a resorbable scaffold and is a great candidate for a protein carrier [9, 10].

The null hypothesis was that the effect of MSNs scaffold and the combination of MSNs scaffold with BMP-2 would not be different from the effect of blood clot on radiographic and histological features during regenerative endodontic treatment of the necrotic immature teeth. The null hypothesis was rejected as MSNs’ subgroups showed better effect than BC subgroups.

Mesoporous silica nanoparticles injectable scaffold was chosen as a therapy regimen in this study, both with and without growth factor (BMP2), because MSNs had revolutionized controlled drug delivery systems. Their beneficial properties, including as well-ordered interior mesopores, resilience, and ease of surface modification, make them suitable platforms for developing multifunctional nanosystems. It contains bioactive chemicals as well as a three-dimensional framework that promotes stem cell growth and differentiation [15, 26].

Because shape is important when developing mesoporous silica-based nanomedicines, we employed rods with large cone-shaped pores (MSR-CP) to load and deliver big protein therapeutics. Furthermore, MCM-41 mesoporous silica was employed because it has a high pore volume, big uniform pore size, and high specific surface area [16].The cone-shaped pores on the surface controlled the immunological response and lowered the pro-inflammatory response of activated macrophages. In addition, BMP-2 loaded MSR-CP accelerated osteogenic differentiation and increased osteogenesis of bone marrow stromal cells [27].

Furthermore, MSNs have unique properties that make them ideal nanocarriers for hosting, protecting, and transporting pharmaceuticals to their target sites. It is possible to insert targeting agents into the exterior surface of MSNs to direct them to sick regions, thereby enhancing specificity and reducing undesirable side effects. Another critical difficulty is to avoid premature cargo release before reaching the destination. In this manner, the pore entrances of MSNs are capped utilizing stimuli-responsive gates. Thus, exposure to internal or external stimuli would cause pore opening and cargo release. Furthermore, multifunctional MSNs can be designed to have synergistic therapeutic effects on sick tissues [28].

The current investigation used 20 µg/ml MS with a total concentration of 100 µg/5 ml, as substantial cytotoxic effects were only seen above 25 µg/ml. There are greater inflammatory reactions above 100 µg [29].

The MSNs scaffold in subgroup MSNs + BMP2 was combined with BMP2 at a protein-to-nanoparticle ratio of 1:10. Proteins are released from mesoporous nanoparticle hydrogels separately via a burst release stage. The delivery of BMP2 using MSNs suspended within a hydrogel carrier overcame the obstacles associated with each method individually [27, 30].

In this study, we chose BMP-2 as the morphogen since it has been shown to play an important function as a biological tool for dentin regeneration [15]. In vitro, recombinant human BMP-2 induces the differentiation of adult stem cells into odontoblast-like cells, boosts their alkaline phosphatase activity, and accelerates expression of the DSPP gene [15]. In vivo, it enhances hard tissue production [31].

Extensive in vivo laboratory research with experimental animals was necessary to demonstrate the efficacy and safety of regenerative endodontics, as certain tests are impossible or unethical to do on human participants [32]. Hence, this is an animal study. Dogs were chosen as the study’s animal model because they are similar to people in terms of apical healing, development pattern, and tooth composition over shorter periods of time (on average one sixth that of humans). Furthermore, they have a high rate of healing, a large number of teeth that might be employed in the study, and access to cavities of appropriate size, all of which would facilitate the research [6, 22, 33].

The Image J software’s TurboReg plug-in was used to standardize radiography examination. This computer application is used to standardize preoperative and postoperative radiographs. The source and target pictures are mathematically aligned using several identical spots on each images [6, 33].

The histology findings in the deposition of hard tissue were identical to the radiography results obtained at both time points. In comparison to MSNs and BC subgroups, MSNs + BMP2 subgroup showed a larger increase in root length, thickness, and decrease in apical diameter. This might be explained by the fact that regeneration employing dentin and pulp-like tissues occurs in MSNs and MSNs + BMP2 subgroup, whereas healing in BC subgroup is assumed to be a reparative process formed by cementum-like tissue and periodontal-like tissue [33].

Regarding the inflammatory cell count, MSNs + BMP2 subgroup in both groups revealed the significant lowest score in comparison to the other experimental subgroups. This might be explained by MSNs which lead macrophages to produce less pro-inflammatory cytokines such as interleukin (IL)-1, tumor necrosis factor, and IL-6. Moreover, MSNs’ decreased capacity to cause inflammation and apoptosis led to downregulation of nuclear factor-κB, caspase 3, and mitogen-activated protein kinases. They also act as an immunogenic sensitizer and the pore shape of Si nanoparticles has a crucial role in their biocompatibility [17]. Furthermore, the injection of BMP-2 dramatically decreased the production of M1 phenotypic markers in M1 polarized macrophages, including IL-1, IL-6, and iNOS, indicating that BMP-2 has a beneficial immune-regulatory effect in an inflammatory environment. Moreover, BMP-2 alone was capable of robustly activating macrophages via the pSmad1/5/8 signaling pathway, increasing angiogenic factor production and hastening osteogenic differentiation of bone marrow stromal cells. According to studies, BMP-2-induced osteogenesis might perhaps be influenced by the neighborhood’s osteoimmune environment [18]. Similar findings were reported before [15, 26].

Both BC and MSNS) subgroups exhibited statistically significantly decreased median inflammatory cell counts without significant difference between them. This could be attributed to traumatized periapical tissues in BC subgroup by excessive instrumentation to cause bleeding and a decreased inflammatory response and apoptosis in MSNs subgroup [16]. Similar inflammatory cell score results were recorded by Wang et al. who reported that revascularization and regeneration techniques cause a minor inflammatory reaction regardless of whether new tissue is formed [15].

As the inflammatory response subsided and the periapical lesion healed, MSNS + BMP2 subgroup in the experimental subgroups had the lowest prevalence of bone resorption score after one and three months. This might be because crucial critical activities during bone regeneration and repair, such cellular differentiation and proliferation, bone matrix mineralization, osteoinduction, and osteogenesis, can all be triggered or increased by utilizing nanosized and nanostructured Si-rich materials [19].

MSNs alter the osteoblast/osteoclast ratio by increasing pro-osteoblastic activity and mineralization, encouraging osteogenic differentiation and angiogenesis, suppressing osteoclasts, and modifying particular molecular complexes that regulate bone homeostasis [34]. Furthermore, including osteoinductive proteins (BMP2) [35] and related encoding peptides [36] or encoding plasmids [35, 37] into MSN-based formulations can aid or accelerate bone repair. These results agreed with those of a previous study despite the fact that they noted that some revascularization samples could exhibit symptoms of bone resorption up to three months postoperative [38].

Regarding presence of vital tissues within the pulp space after one- and three-month evaluation periods, MSNs + BMP2 subgroup demonstrated significat higher score than BC subgroup. This might be explained by the fact that MSNs perform as an effective scaffold, allowing growth factor (BMP2) laden cells to be released over a longer period of time, with improved cell organization and diffusion compared to the BC subgroup. The most plausible tissue in-growth mechanism is the release of ions from the disintegration of MSNs scaffolds, which promotes angiogenesis and osteogenesis. In vivo investigations showed that MSNs containing BMP-2 improved bone regeneration performance [27, 39]. All of these findings hint to the potential advantages of using mesoporous silica as a scaffold for dentin and dental pulp engineering.

The newly generated tissue in BC subgroup after one month has a structure comparable to that of the periodontal structure. The freshly produced hard tissue is comparable to cementum in that it contains cementocyte-like cells, has a fibrous link to the surrounding connective tissue, and adheres directly to the dentin. Blood clots are useful because they offer the developing bacterium with the nutrients it requires to thrive. These findings are consistent with those of earlier workers who found cementum accumulation in the apical third after revascularization [1, 15].

In MSNs and MSNs + BMP2 subgroups after one month, statistically significant higher-level tissue in-growth inside the canal was noticed, reaching the middle third. These findings corroborated those of earlier authors who stated that growth factors or nano scaffold components are mixed into nano scaffold materials to construct smart scaffolds for tissue engineering of injured hard tissues [8, 41].

At three months, MSNs + BMP2 subgroup demonstrated a significant higher incidence of apical closure than BC and MSNs subgroups. As a result of the apex’s approach with newly deposited hard tissue, samples showed biological apical closure. These results are in agreement with the results of Thibodeau et al. [1].

Recent breakthroughs in nanotechnology have made it possible to tackle a variety of infectious illnesses with fewer negative side effects by utilizing already-available Nano Carriers (NCs). To successfully remove intra- and extra-radicular infection, microbial biofilms must be combated even in canal regions inaccessible to chemomechanical debridement, as well as penetrate deeply into dentinal tubules. It is unable to make definite conclusions regarding the best NCs for obtaining efficient antimicrobial results, even in endodontics, due to the intrinsic constraints of various methodological approaches and stages of NC development. This framework calls for future study into the use of several classes of NCs to neutralize tissue debris, promote total root canal system disinfection, prevent re-infection, and quantify sterility hold time. To do this, it is critical to emphasize professional NCs that promote root sterility, improve dentin matrix mechanical integrity, and have a preference for endodontic microbiological spectrum. These NCs must also be able to penetrate deep into the radicular dentine tubules [42].

Mesoporous Silica is a Smart Nano Carrier (MSSNC) that is employed because to its characteristics (bioactive loads are packed into the high-capacity pores, and their discharge may be triggered by a variety of stimulus-responsive molecular gatekeepers). This resulted in MSNs scaffolds producing outstanding outcomes when compared to BC regenerative endodontic therapy. MSSNCs may respond to both exterior (light and magnetic sources) and internal stimuli (pH, redox, enzyme, chemical, temperature, and biomolecules). The drug release effectiveness of MSSNCs is largely reliant on mesopore width and volume, as well as the chemical properties of the functionalized surface [42].

There are various MSNCs that function as specialized composite drug carriers. Weldrick et al. have tested a smart active nanocarrier containing penicillin G and oxacillin. The scientists found that the formulation revealed a significant improvement compared to the similar solo antibiotics, decreasing the viable germs cells to ∼ 6 log CFUmL − 1 (99.9999%) in planktonic suspensions and no living cells were observed in biofilms [43].

Nanomaterials’ widespread use in medicine allows for the development of sophisticated drug delivery systems with regulated drug loading efficacy, biodistribution, cell/tissue targeting, therapeutic actions, cytotoxicity, selectivity, imaging capability, blood circulation time, half-life, and excretion. It is often assumed that all of these properties of nanomaterials are completely determined by their surface chemistry, total surface area, hydrodynamic size, drug loading, and so on. The phenomenon of nanomaterial form is usually investigated in connection to blood circulation time, biodistribution, and systemic toxicity [44]. Nonetheless, multiple studies have shown its effects on the biological functioning of human and microbial cells. These functions include shape-induced directed differentiation [45], cellular death via apoptosis [46], necrosis [47], gene transfection and transfer [48], metabolic modification [49], and other activities. The delivery of NPs to the cell surface, as well as their interaction with cellular structures and possible subsequent response, is heavily influenced by various parameters such as (a) the physicochemical properties of the NPs, (b) the cell and tissue type, and (c) the intracellular fate of the NPs in the various organelles, including biopersistence, exocytosis, and/or transfer to other cells [50]. More studies give compelling evidence that nanostructures not only passively interact with cells, but also actively engage and mediate the molecular processes required to regulate cell activities [47]. Attachment, spreading, proliferation, signaling, and differentiation are all cellular processes that rely on nanomaterial-cell interactions. These materials are designed to operate as an artificial extracellular matrix (ECM), containing a combination of chemical, mechanical, physical, and biological components that provide the necessary signals to govern the fate of cells [51].

## Conclusions

The MSNs with/without BMP-2 scaffolds enable the continuing growth of roots in teeth with necrotic pulps and periapical pathosis. Therefore, both scaffolds are successful alternatives to blood clot therapy during regenerative endodontic treatment of immature teeth with necrotic pulps and periapical pathosis. Addition of BMP-2 to MSNs scaffold improved its outcome and thereby reduced adverse consequences in regenerative endodontics.

### Electronic supplementary material

Below is the link to the electronic supplementary material.

Supplementary Material 1

## Data Availability

The datasets used and/or analyzed during the current study are available from the corresponding author upon reasonable request.

## Abbreviations

ATR: Attenuated total reflection
BC: Blood clot
BMP2: Bone morphogenic protein
DSPP: Dentin sialo-phosphoprotein
ECM: Extracellular matrix
EDX: Energy dispersive X-ray
FTIR: Fourier-transform infrared
MCM-41: Mobile Composition of Matter No 41
MSNs: Mesoporous silica nanoparticles
MTA: Mineral trioxide aggregates
Sio2: Silicon dioxide
STEM: Scanning transmission electron microscopy
TEOS: Tetraethyl orthosilicate
