# Supplementary material for: Regenerative potential of mesoporous silica nanoparticles scaffold on dental pulp and root maturation in immature dog’s teeth: a histologic and radiographic study
Source: BMC Oral Health. 2024 Jul 18;24:817. doi: 10.1186/s12903-024-04368-6 (PMC11264670; doi:10.1186/s12903-024-04368-6)
Supplement: Supplementary file 1 — Supplementary Material 1 [file 12903_2024_4368_MOESM1_ESM.docx]

- **Illustrative figure showing that the cuts in fig 3 is at the periapical areas**


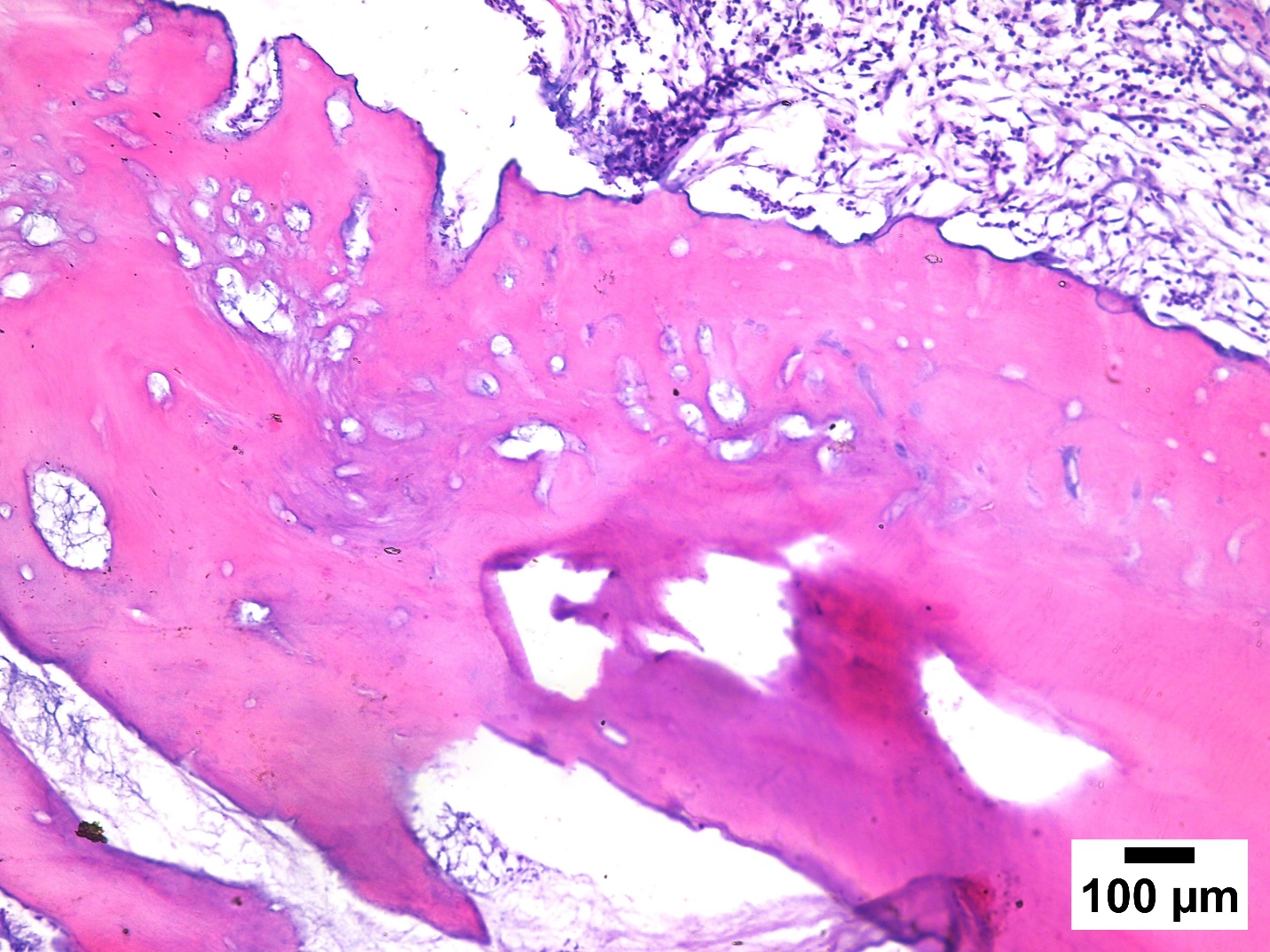


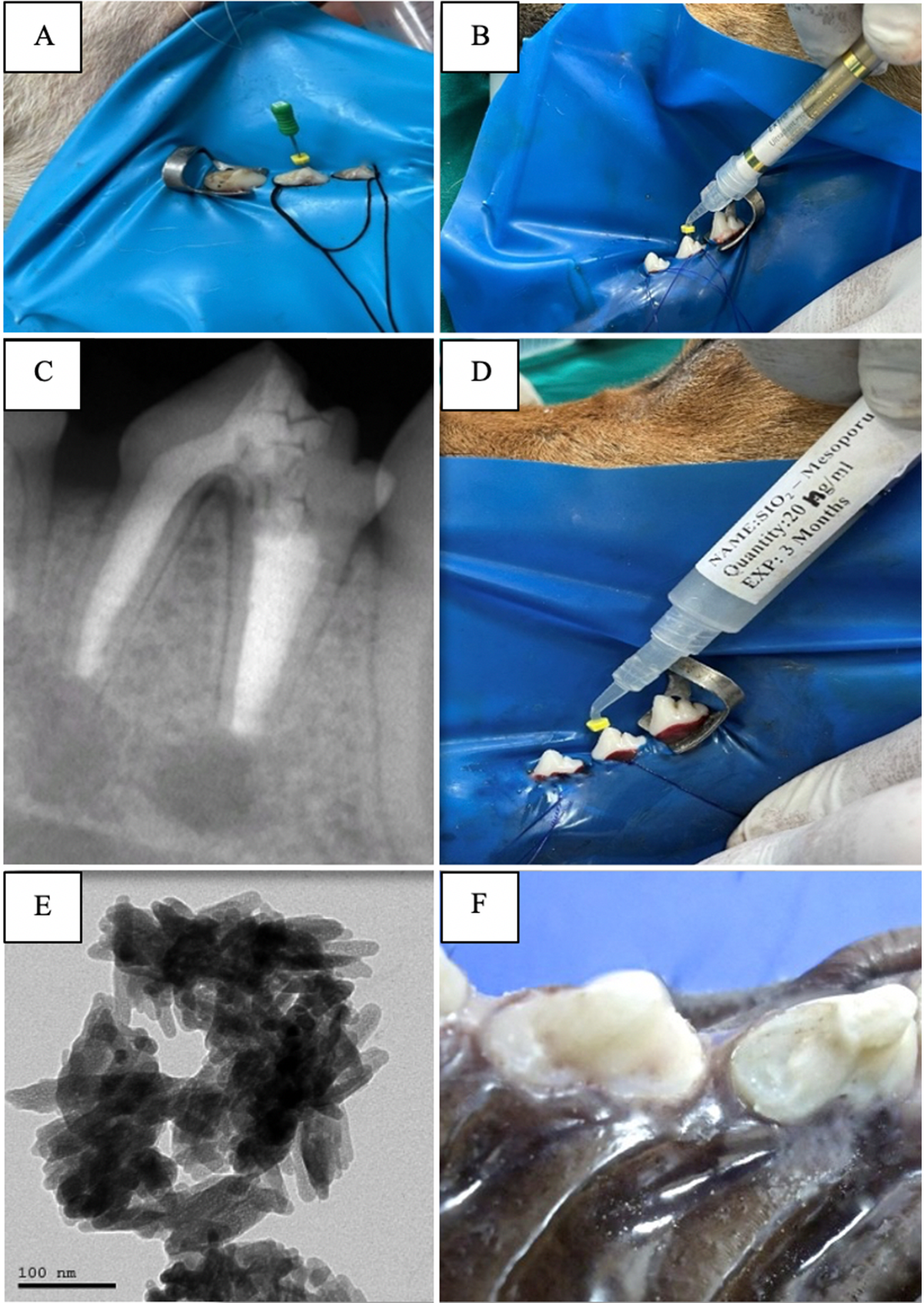


- Figure showing procedural steps
- A: Showing a sterile file size #35 was used to disrupt the remnant pulp tissue in the canal
- B: Showing injection of calcium hydroxide
- c**:** Radiograph after calcium hydroxide placement
- D: Showing injection of Mesoporous silica Nanoparticle scaffold
- E: Transmission electron microscopy images showing rod like shape of MSNs (100nm)
- F: Showing access cavity restored using glass ionomer restoration.
